# Implications for fault locking south of Jakarta from an investigation of seismic activity along the Baribis fault, northwestern Java, Indonesia

**DOI:** 10.1038/s41598-022-13896-6

**Published:** 2022-06-16

**Authors:** S. Widiyantoro, P. Supendi, A. Ardianto, A. W. Baskara, C. A. Bacon, R. Damanik, N. Rawlinson, E. Gunawan, D. P. Sahara, Z. Zulfakriza, Y. M. Husni, A. Lesmana

**Affiliations:** 1 Global Geophysics Research Group, Faculty of Mining and Petroleum Engineering, Institut Teknologi Bandung, Bandung, 40132 Indonesia; 2 Faculty of Engineering, Maranatha Christian University, Bandung, 40164 Indonesia; 3 Agency for Meteorology, Climatology, and Geophysics (BMKG), Jakarta, 10720 Indonesia; 4 Department of Earth Sciences, Bullard Labs, University of Cambridge, Cambridge, CB3 0EZ UK; 5 Geophysical Engineering Study Program, Faculty of Mining and Petroleum Engineering, Institut Teknologi Bandung, Bandung, 40132 Indonesia; 6 PT. Reasuransi Maipark, Multivision Tower, Menteng Atas, Jakarta, 12960 Indonesia

**Keywords:** Solid Earth sciences, Seismology, Tectonics

## Abstract

Recent borehole seismic deployments conducted along the Baribis Fault in northwestern Java reveal that it may be active. In this study, we exploit these data to locate proximal earthquakes using a relative relocation technique, estimate their moment magnitudes using a spectral fitting method and compute their focal mechanisms via waveform inversion. We observe that seismicity in the eastern part of the fault is significantly higher than in the west, where a previous GPS study of the region south of Jakarta demonstrated the existence of high compression rates. These observations imply that the western Baribis Fault is locked, and that neighbouring areas, including southern Jakarta and its surroundings, may be highly vulnerable to future sizeable earthquakes when accumulated elastic strain energy is eventually released during fault rupture. Significantly, the current generation of Indonesia’s national hazard maps have not considered seismicity along the Baribis Fault. Our new results therefore call for an urgent reappraisal of the seismic hazard in northwestern Java that carefully takes into account the Baribis Fault and its earthquake potential, particularly in light of its proximity to Jakarta, a megacity that lies at the heart of one of the most densely populated islands in the world.

## Introduction

**Figure 1:**
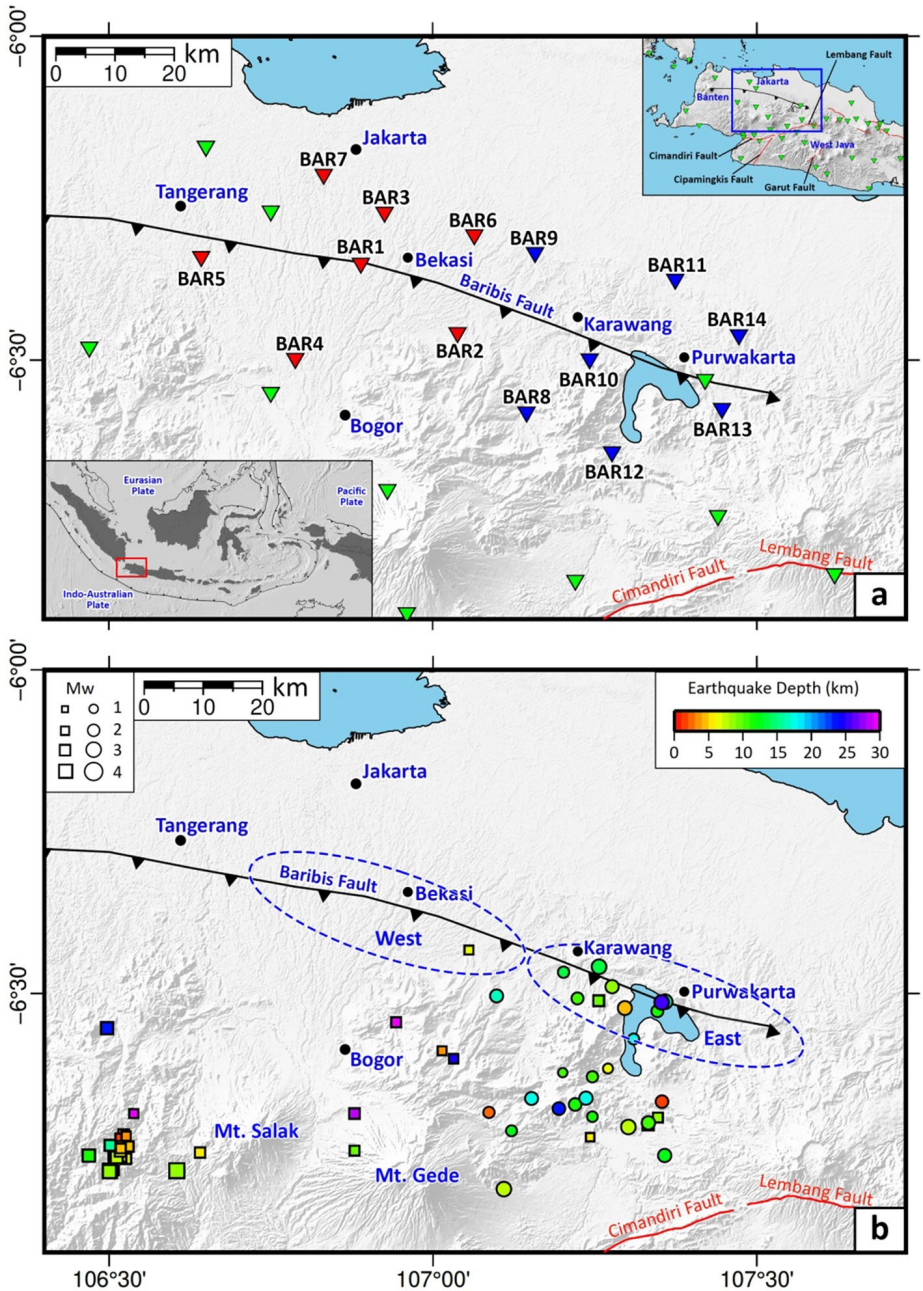
Regional setting and distribution of epicenters. (**a**) Map showing the location of the study area. Blue inverted triangles depict the location of borehole seismic stations used in this study. Red inverted triangles denote the borehole seismic stations used in the previous study by Damanik et al.^18^. Inverted green triangles represent Indonesian Agency of Meteorology, Climatology and Geophysics (BMKG) stations used in both studies. We moved stations BAR1-BAR7 to stations BAR8-BAR14 in September 2020. The black line with a sawtooth represents the Baribis Fault, the location of which was extracted from Simandjuntak & Barber^8^. The lower left inset shows the location of the western part of West Java (red rectangle) with respect to the Indonesian region. Upper right inset shows the location of the study area (blue rectangle) with respect to the western part of West Java. Red lines correspond to other major crustal faults including Cimandiri and Lembang Faults^3,4^, Garut Fault^13^, and Cipamingkis Fault^14^. (**b**) Map of the relocated earthquakes from July 2019 to July 2021. Events were determined and relocated using borehole and BMKG seismometers when they were recorded by both instrument types, otherwise by borehole seismometers only (see Supplementary Table 2). The colored squares and dots depict epicenters of events from July 2019 to August 2020^18^ and from September 2020 to July 2021 (this study), respectively. Dashed ellipses depict the western and eastern parts of the fault discussed in this paper. The Generic Mapping Tools (GMT) version 6.0^59^ (https://www.generic-mapping-tools.org/) was used to make this figure.

Major cities in northwestern Java, such as Jakarta, Tangerang, Bekasi, Karawang and Purwakarta (Fig. 1a) are underpinned by a geologically complex terrane that ultimately arises from the convergence of the Australian Plate and the Eurasian Plate along the Java Trench. In this region, the Australian Plate subducts into the upper mantle at a steep angle^1–5^ and it is earthquakes that occur along the associated subduction megathrust that pose the greatest danger to nearby cities. However, these cities are also subject to earthquakes generated by active crustal faults that traverse regions with high population densities. These faults include the Cimandiri Fault^6,7^, Baribis Fault^8–10^, Lembang Fault^6,11,12^, Garut Fault^13^, and Cipamingkis Fault^14^.

Marliyani et al. (2016)^15^ propose that the Cimandiri Fault is divided into six main segments, i.e. Loji, Cidadap, Nyalindung, Cibeber, Saguling and Padalarang. These segments have previously ruptured to produce earthquakes that vary in magnitude from Mw 6.5 to 6.9. At the eastern end of the Cimandiri Fault, the 29 km left-lateral Lembang Fault can be found^6,11^. Daryono et al.^12^ estimate that it has a slip rate of ~ 3.45 mm/yr and is capable of generating earthquakes up to Mw 7.0. Another active fault in the region is the Cipamingkis Fault, which is located at the southern end of the Cimandiri Fault with a SW-NE strike^14^. Seismicity recorded in 2018 suggests that this fault is active, but its slip rate and geological segmentation are not yet known. Furthermore ~ 80 km to the east of the Cipamingkis Fault, there is an active fault (Garut Fault) of estimated length ~ 36 km^13^. Both the Cipamingkis and Garut Faults are newly identified and require further information on their fault parameters.

In the case of the Baribis Fault, the focus of our study (see Fig. 1a), Aribowo et al.^10^ map fault lineaments in unprecedented detail using morphotectonics with the aid of an 8 m resolution digital elevation model. Together with analysis of subsurface geophysical data that include seismic reflection and resistivity imaging, they conclude that this southerly dipping fault has been active during late Pleistocene to Holocene times until at least 7 ka. Nguyen et al.^16^ use OpenQuake software to determine the most plausible source of a number of historical earthquakes in Indonesia that have reports of damage and ground shaking. They find that an earthquake of between Mw7.0–8.0 likely occurred on the Barbiris Fault on January 22, 1780 followed by a second event of between Mw7.0–7.7 on October 10, 1834. The presence of this fault has also been verified by a number of other studies, including that of Simandjuntak and Barber^8^ from structural/geological mapping using seismic reflection data from on-shore exploration^17^ and Koulali et al.^9^ from regional GPS work. In the latter case, elastic and viscoelastic modelling are used to suggest that the Barbiris Fault may accommodate up to 5 mm/yr of convergence between Java and the Sunda Block. However, none of the above studies found any evidence of recent earthquake activity.

This study extends our previous work on earthquake monitoring along the Baribis Fault that involved the deployment of borehole seismographic stations around Jakarta^18^. Previously, the status of the Baribis Fault as an active or inactive fault was a matter of considerable debate^9^. While the results of Damanik et al.^18^ suggest that the Baribis Fault is seismically active, it relied on the detection, location and characterisation of only two events that appeared to be fault related. In this study, additional earthquake monitoring was carried out via the installation of a further seven borehole seismometers around the Baribis Fault near Karawang and Purwakarta (Fig. 1a; Supplementary Table 1). Here we aim to determine in more detail the seismic activity of the Baribis Fault and explore whether it may pose a threat to nearby population centers. Our results will be important for future updates of the current hazard maps of Indonesia, which have not considered the Baribis Fault due to insufficient data^3,4^.

## Results

The new data acquired by deploying seven borehole seismometers, which are notably more sensitive to small events compared to the existing BMKG surface network^18^, allow us to address fundamental scientific questions about the Baribis Fault, including its level of activity, whether ongoing slip is spatially variable, and its risk potential for nearby population centres. Our new observations combined with the results of our previous borehole experiment^17^ indicate that in total 12 detectable earthquakes occurred very close to the Baribis Fault line during the last ~ 2 years (July 2019–July 2021) of deployment (Fig. 1b). Of the 12 events, 10 were detected in this study, with the remaining two by Damanik et al.^18^; this underscores the value of the new dataset in understanding seismicity generated by the Baribis Fault.

Estimated moment magnitudes for the complete set of 61 events are shown in Supplementary Table S2, and exhibit a range between Mw 1.9–4.3. For the events we associate with the Baribis fault (Supplementary Table S3), the magnitude range is Mw 2.3–3.1. The magnitude distribution as a function of time does not suggest that any subset of the events generated by slip on the Baribis Fault is likely to represent a (foreshock)-mainshock-aftershock sequence.

**Figure 2:**
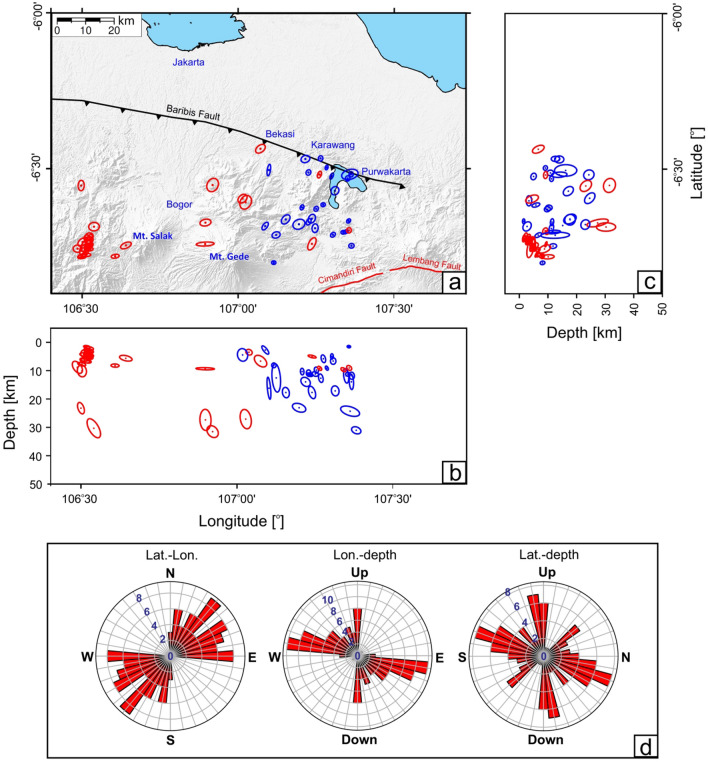
Bootstrap uncertainty analysis results. Map of the relative location errors for the relocated events shown in Fig. 1b. (**a**) map view. (**b**) and (**c**) vertical cross sections in longitude and latitude, respectively. They are plotted on a 1 to 1 horizontal to vertical scale. Location error estimates are represented by 95% confidence ellipsoids obtained from a bootstrap analysis of the final double-difference vector. Red and blue dots depict hypocenters from Damanik et al.^18^ and this study, respectively. (**d**) Rose diagrams of the event uncertainties, as defined by the major axis of the 95% confidence ellipsoids. From left to right: map view, longitude vs. depth, and latitude vs. depth. Numbers in the diagram indicate the number of events within each bin of 10 degrees. The Generic Mapping Tools (GMT) version 6.0^59^ (https://www.generic-mapping-tools.org/) was used to make this figure.

Figure 2a–c shows the relocated seismicity obtained by applying hypoDD to 61 on-land events, along with associated error ellipsoids estimated from a bootstrap method. In general, the location uncertainties are small with average errors of 2.4 and 2.7 km in the horizontal and vertical directions, respectively, thus allowing a reasonably detailed interpretation of the distribution of seismicity. Rose diagrams (Fig. 2d) of the azimuths of the major ellipsoid indicate that the peak horizontal uncertainties are in the E-W (almost parallel to the Baribis fault) and NE-SW directions. Based on the rose diagrams that include a vertical component, the distribution of major ellipsoids tends to be balanced between the horizontal and vertical directions with a slight dominance in the vertical direction.

**Figure 3:**
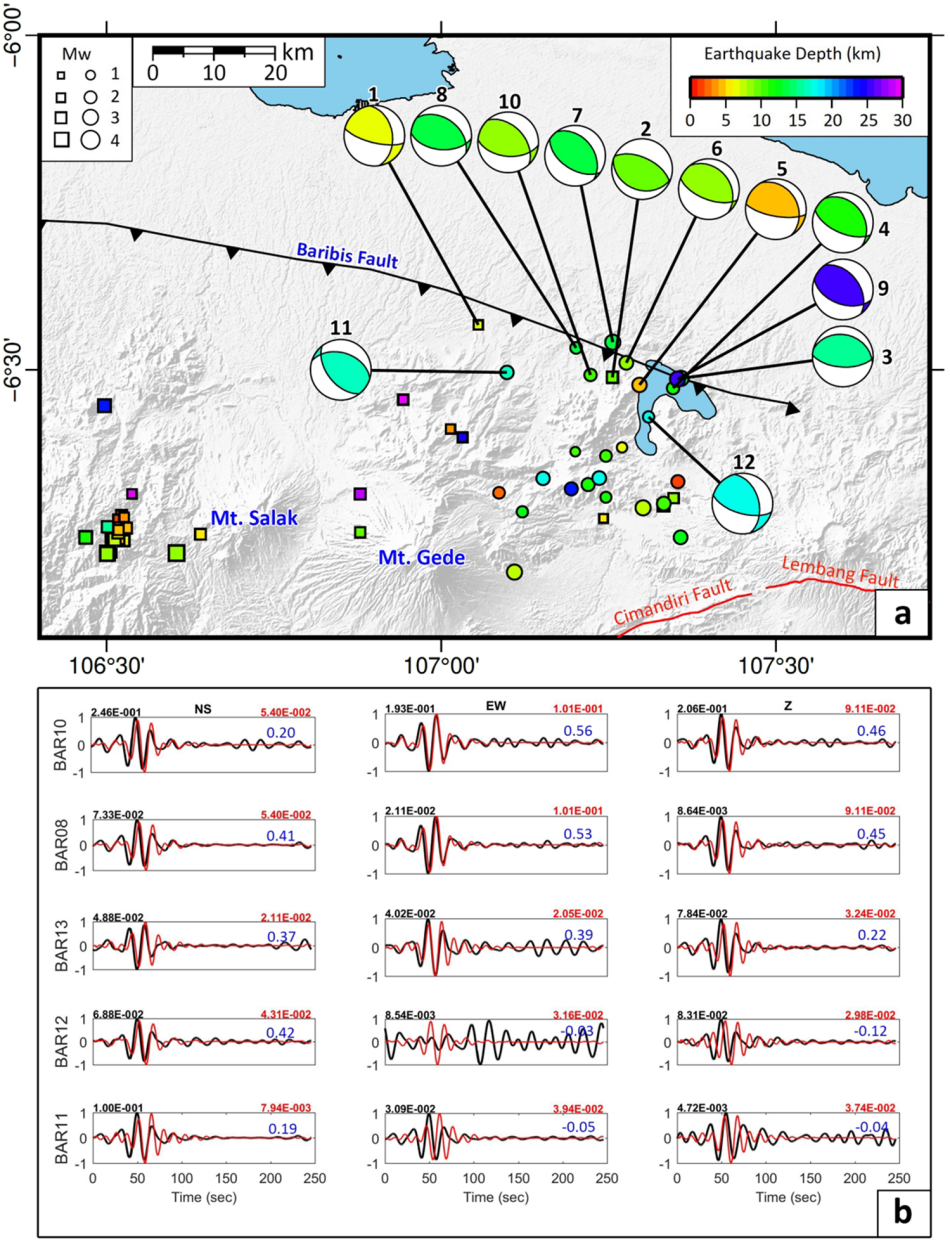
Focal mechanism analysis results. (**a**) Focal mechanism solutions for the earthquakes that occur along the Baribis Faults; two from Damanik et al.^18^ (event no. 1–2, squares) and eight from this study (event no. 3–10, dots). (**b**) Example of three-component waveform inversions at stations BAR08, BAR10, BAR11, BAR12, and BAR13 for event no. 4 in (**a**) that occurred on 11 February 2021. Black and red waveforms are observed and synthetic waveforms, respectively. Blue numbers denote variance reduction that characterizes the match between the observed and best-fitting synthetic data. The black and red numbers above the panel of the waveform fitting are the peak amplitudes of observed and synthetic waveforms respectively. The y-axis denotes the normalized displacement. Note that events no. 3, 4, 8, 9, and 10 were recorded by borehole stations only. The rest were recorded by borehole and BMKG stations. The set of waveform fitting results for the remaining events are shown in Supplementary Figs. S10–S20, while the data acquisition plot for the first year and second year of the borehole station deployment is shown in Supplementary Fig. S21. The Generic Mapping Tools (GMT) version 6.0^59^ (https://www.generic-mapping-tools.org/) was used to make this figure.

We also performed a focal mechanism analysis of the 12 events that we suggest occurred along the Baribis Fault to characterise the nature of the fault slip using the ISOLA package^19^, with waveforms filtered between 0.04 and 0.09 Hz. The resulting focal mechanism solutions determined using both borehole and BMKG data or just by borehole data for events that were not recorded by the BMKG stations, are mostly consistent with a slightly oblique thrust fault (see Fig. 3), and the similarity of the solutions suggesting that all the earthquakes are likely caused by slip on the same fault surface. This fault has an average strike of 109^o^E and an average dip of 57°S (see Supplementary Table S3). The strike and dip of the fault support the previous finding by Simandjuntak and Barber^8^ who mapped the fault as striking almost E-W and dipping southward.

## Discussion

The benefit of including borehole data in monitoring seismic events along the Baribis Fault was discussed in detail by Damanik et al.^18^, in which the borehole data are more sensitive to small events compared to the existing BMKG surface network that suffers from anthropogenic noise. However, since most of the events occurred some distance away from the borehole array, we combined our borehole data with data collected by existing BMKG stations during the same recording period to better constrain hypocenter locations. This combined data set enables us to locate events along the Baribis Fault with more confidence (see Fig. 2). In particular, it is shown that the eastern segment of the fault is significantly more active than the western segment (Fig. 1b), a result that is clearly not an artifact of station distribution. Damanik et al.^18^ only recorded two events along the Baribis Fault during one year of their borehole experiment, whereas we recorded 10 events that we attribute to motion along the eastern segment of the fault. This significant addition of earthquakes greatly reinforces the argument that the Baribis Fault is active. We note that the region immediately south of the Baribis Fault contains active volcanoes (Mt. Salak and Mt. Gede), which are the most likely source of the earthquakes not associated with the fault. The hazard associated with these phenomena are quite well known, hence our focus on the earthquake potential of the Baribis Fault, which is poorly understood and not considered in the current national hazard map of Indonesia.

Our analysis of the focal mechanisms of events along the Baribis Fault (Fig. 3) consistently points to slightly oblique thrust events. We interpret the nodal plane of the focal mechanisms based on the results of previous geological studies^8^ that conclude that the Baribis Fault is dipping southward. The result of our analysis of the focal mechanisms, shown in Fig. 3A, indicates that the fault has an average dip of 57° S. Several of the focal mechanism solutions suggest a minor strike-slip component, which is mainly dextral (right lateral), and may be due to movement along the Great Sumatran Fault^20^.

As noted earlier, Koulali et al.^9^ estimated that the Baribis Fault may accommodate up to 5 mm/yr of convergence between Java and the Sunda block; if this were the case, then the corresponding strain rate and maximum likely earthquake magnitude is of interest. While the relationship between tectonic strain, slip rate and earthquake moment rate is not unique^21^, by using a fault-scaling relationship between slip rate and earthquake magnitude^22^, we find that for the 5 mm/yr slip rate proposed by Koulali et al.^9^, and a fault length of 100 km, the peak earthquake magnitude would be ~ Mw 7.5 (equivalent to 2.2 × 1e20 Nm). Using the relationship of D’Agostino^23^, we have that *M* = *2μHAε*, with *M* = 2.2 × 1e20 Nm, *μ* = 3 × 1e10 N/m^2^ (Gunawan et al.^24^; Barbot^25^), *H* = 15 km (Koulali et al.^9^), *A* = 5000 km^2^ (Gunawan and Widiyantoro^14^), such that the strain is *ε* = 4.9 × 1e−5. Assuming the strain accumulates for 800 yr, we find that the strain rate is 61 nstrain/yr.

The lack of seismicity in the western segment of the Baribis Fault is very intriguing. When sufficient geodetic and seismic data are available, it is possible to analyse the relationships between localized deformation and earthquake activity^26,27^. In general, seismic gaps (regions with fewer earthquakes that all tend to be small in magnitude) or regions that are locked, are associated with high slip deficits (e.g. when fault slip is hampered by increased friction^28^). However, it is noted that the presence of a seismic gap may also be caused by slow slip events, which could be responsible for ongoing energy release^29,30^. In the case of Jakarta, however, previous studies by Musson^31^, Albini et al.^32^ and Nguyen et al.^16^ found evidence from historical data that major buildings in Batavia (now Jakarta) were destroyed by destructive earthquakes in 1699, 1780 and 1834, with the last two events likely associated with the Baribis Fault. Why the western part of the Baribis Fault is seismically less active than the east, as discovered in this study, may be related to the pattern of strain rates determined using Global Positioning System (GPS) velocity data by Gunawan and Widiyantoro^14^.

**Figure 4:**
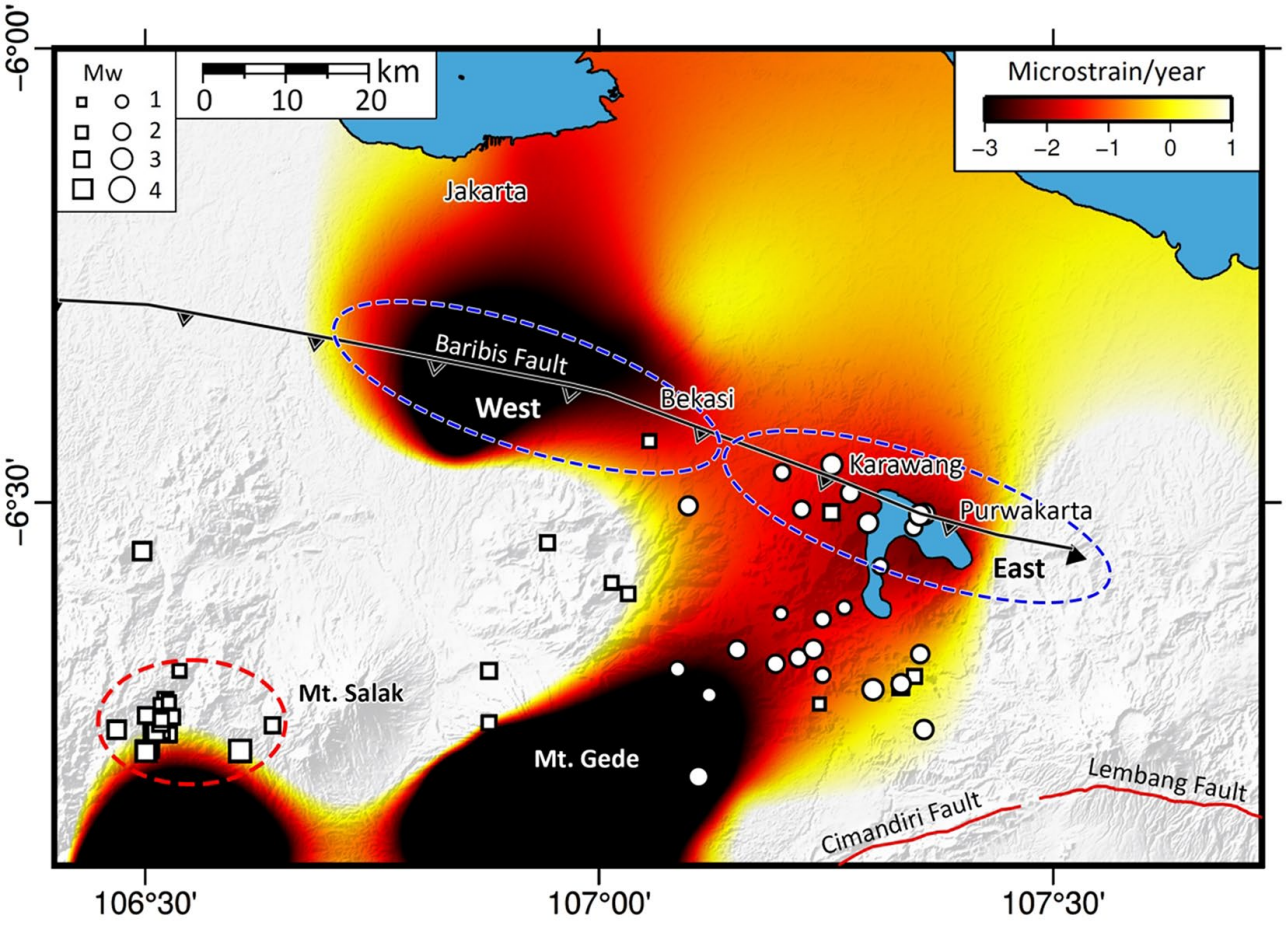
Map of dilatation rate based on Gunawan & Widiyantoro^14^ for the study region. Positive and negative values indicate extension and compression, respectively. Squares and dots represent epicenters of relocated events recorded by the borehole seismometers used by Damanik et al.^18^ and deployed in this study, respectively. Note that the western part of the Baribis Fault has a high compression rate and fewer earthquakes compared to the east, which may be related to fault locking. The dashed ellipse depicts the location of a swarm in the study area (see also Sianipar et al.^34^ and Supendi et al.^35^). The Generic Mapping Tools (GMT) version 6.0^59^ (https://www.generic-mapping-tools.org/) was used to make this figure.

Like Widiyantoro et al.^24^ who superimpose the epicentral distribution of earthquakes on a slip deficit model to examine possible seismic gaps south of Java, we combine the GPS-derived strain rate model of Gunawan and Widiyantoro^14^ and the earthquake locations from our borehole experiments in Fig. 4 to investigate possible locked areas along the Barabis Fault. Widiyantoro et al.^24^ infer that where slip deficit is high, there is a correlation with seismic gaps, and hence these areas are likely to be sources of future strong earthquakes. In this case, we interpret that the high compression rate in the region to the south of Jakarta may be related to a locked area in the western part of the Baribis Fault, which exhibits fewer events (see Figs. 1b and 4). Why this region appears locked is unclear; possibilities include changes in the frictional properties of the fault, the orientation of the stress field and the N-S change from compression to expansion (Fig. 4), a phenomenon that has previously been associated with a lack of seismicity^33^. We also acknowledge that high strain accumulation may not only be related to locked faults during interseismic periods but also to volcanic activity^14,34,35^ and swarm activity^36^. In the southwestern region, as noted previously, there are two active volcanoes, Mt. Gede and Mt. Salak. Supendi et al.^37^ analyzed the August 2019 events and suggest that the earthquakes which occurred at around 106.5°E and 6.7°S are related to volcanic activity. Furthermore, Sianipar et al.^36^ show that the earthquakes were initiated by fluid intrusion into the seismogenic zone, while stress changes from the largest event affected the evolution of the swarm (see Fig. 4).

We note that the results of this study are based on a relatively small number of earthquakes of Mw < 4.3 recorded over a two-year period. It is possible that this modest sample of events is not representative of the longer-term slip behaviour of the Baribis Fault, and as such further work is needed (e.g. a denser deployment of borehole stations for a longer period of time). However, the current study does bring into focus the potential for large terrestrial earthquakes to occur near Jakarta in future if a locked zone of the fault is indeed present and ruptures after accumulating significant amounts of elastic strain energy. It is also crucial to carefully include the active Baribis Fault in any update of existing Indonesian hazard maps, since this will ultimately feed into disaster management planning and building design.

## Materials and methods

### Borehole seismometer array

A temporary installation of seven borehole seismometers was completed in August 2020, as a continuation of the collaboration between Institut Teknologi Bandung (ITB) and PT. Resuransi Maipark Indonesia^18^. The instruments were previously installed in and around Jakarta for one year, and for the current study, they were moved eastwards to record events for one year from September 2020 to July 2021 (see Fig. 1a and Supplementary Table S1 for their detailed locations). The borehole array design consists of three-component (3C) C100 wideband seismometers deployed at a depth of between 8 and 11 m below ground level using a PVC pipe and a Sri32L Geobit digitizer placed on the surface in a compact shelter 1 m^3^ in size (Supplementary Fig. S1). The seismometers have a flat response between 10 s and 98 Hz, a sensitivity of 1500 V/m/sec on all components, and the signal is digitised at 32 bit resolution. In this study, a sample rate of 100 sps is used, which is the same as that used by Damanik et al.^18^. The drilling of the borehole was assisted by a digger machine, and the mini shelter was made of iron to protect the instruments from theft or damage (Supplementary Fig. 1). The borehole seismic stations were powered by 12 Volt 75 Ah lead-acid car batteries, which were replaced approximately every 1.5 months. Examples of 3C seismograms recorded by the borehole stations are shown in Supplementary Figs. S2–S4.

### PPSDs

We assessed the quality of the seismic data by evaluating the average of the recorded noise level through computing a probability density function (PDF) and plotting the probabilistic power spectral density (PPSD) (Supplementary Fig. S5). We removed the mean, detrended and then deconvolved the instrument response of the seismogram data without filtering. The signal was then windowed using the Hanning tapered window function with a window length of 10 min to make sure that each of these windows did not overlap one another. The PPSD was calculated using a standard FFT algorithm, and the results were interpolated logarithmically. The PPSD results from all windows at each station were then stacked. The PDF was calculated using an adaptive kernel density estimation algorithm based on a linear diffusion process^38^. The PDF construction process was conducted iteratively to obtain the optimal bandwidth by considering the most common linear diffusion with the same stationary density as the estimated pilot density. The program for calculating PDFs with adaptive kernel density estimates was taken from Botev^39^ and calculations were performed using the vertical component seismograms (BHZ) only.

### Earthquake relocation

Data processing was conducted through two stages as in Damanik et al.^18^: (i) event identification using the FilterPicker Algorithm^40^, (ii) enhanced automated detection using Quakemigrate^41^ to detect any small events that may have been missed by FiilterPicker and (iii) manual picking of P- and S-wave arrival-times of 3C waveforms using Seisgram2K^42^. The FilterPicker Algorithm has two stages of evaluation to determine the arrival time, with each stage having a triggeringAlgorithm^40^, (ii) enhanced automated detection using Quakemigrate^41^ to detect any small events that may have been missed by FiilterPicker threshold defined by parameters S1 and S2, respectively. For the first stage, a trigger is declared when the value of the characteristic function (obtained from a recursive multi-band filtering scheme) is larger than S1, at which point the trigger time is stored and will be used in the second evaluation stage. The second evaluation stage is carried out to minimize false positives due to the presence of spikes in the seismogram. This process involves integrating (or summing) several sample points of the characteristic function value after the trigger time, with the integral then compared to S2. Both of these threshold values must therefore be exceeded in order for the arrival time picking to be triggered. The standard values of the S1 and S2 parameters from Lomax et al.^40^ are both equal to 10. Vassallo et al.^43^ attempted to refine these values and found that S1 = 9.36 and S2 = 9.21 appear to be optimal. In this study we used values of 8 and 8 to reduce the probability of false negatives.

Picking is carried out when at least three stations are triggered within a certain time span. In this case, we set the time span as 10 s, meaning that if at least three stations are triggered in this time span, we attempt to pick these arrivals as though they emanate from the same event. This process still produces a lot of false positives on account of the small threshold values we use for S1 and S2. However, every detected event is checked manually to ensure accurate arrival time picking and to eliminate false positives.

In an attempt to improve on the FilterPicker event detection results, we applied a very different automatic detection approach based on waveform migration and stacking, as encapsulated in the QuakeMigrate software^41^. In this approach, STA/LTA (short-term-average/long-term-average) functions are generated from continuously recorded waveforms; the peaks of these functions and their width correlate with phase arrive times and their associated uncertainty. These functions are then aligned via a grid search over candidate locations using a pre-calculated traveltime look-up table, with peaks in the stacked coalescence function illuminating the location and origin time of possible events, which are accepted as real if they exceed a dynamic threshold based on a variable multiplied by the median of the data within a 300 s window (see Supplementary Figure S6 for an example of how an event within the array is detected by QuakeMigrate). In this study, we detected a total of 112 events using QuakeMigrate, of which 24 lie in our study region that were not previously detected using FliterPicker. Of these 24 events, two are located ~ 10 km south of the eastern segment of the Baribis Fault, 14 are located in the Bogor Swarm area, and the rest elsewhere in the study region. All events detected by QuakeMigrate were manually assessed, and the arrival times of the 24 events in the study area were picked, and used in subsequent analysis.

The earthquake hypocenters were determined using the Hypoellipse software^44^. To check the reliability of the hypocenter solutions, a Wadati diagram of arrival times was plotted (see Supplementary Fig. S7). We relocated the hypocenters from this study together with those from Damanik et al.^18^ using HypoDD^45^ For each event, P and S arrival-time catalog data were searched to identify paired events with similar travel-times. The hypocentral separation maximum was set to 20 km, the maximum number of neighbours per event was set to 10, the minimum number of links needed to define neighbours was set to 8 and a maximum distance between cluster centroid and station was set to 100 km. Extensive testing of input parameter choices was done to determine the combination of values that achieved the optimum results, although the final distribution of relocated hypocenters does not change significantly across a reasonable range of input values. A comparison of epicenters and travel-time residuals before and after relocation are shown in Supplementary Figs. S8 and S9, respectively.

We estimated the location uncertainty (see Fig. 2) using a bootstrap method^46–49^. Using the final hypocenters, samples were randomly drawn (with replacement) from the full set of observed residuals and substituted for each measurement; events were then relocated with the re-sampled dataset and the resultant location shift analysed. This procedure was repeated 500 times, with error ellipsoids extracted at the 95% confidence level. Gaussian noise with a standard deviation 0.1 s was also added to the data prior to the bootstrap in order to simulate the effect of picking error.

### Moment magnitude

The moment magnitude (Mw) is calculated using the equations of Hanks and Kanamori^50^, taking advantage of the seismic moment estimated from fitting the displacement spectrum amplitude using a Brune type model^51,52^. We adopted the grid search technique to obtain low frequency spectral level, corner frequency, and rock quality factor that can provide the best fit to the displacement spectrum amplitude data. We independently calculated the magnitude of the P wave (in the vertical component) and the S wave (in the transverse component) for all stations. We set the same window parameters for the P and S waves, i.e., 0.5 and 2.0 s before and after the arrival time, respectively. A constant average radiation pattern correction for the P wave (0.44) and S wave (0.60) was used, utilizing previous results^53^. The final moment magnitude, which represents the strength of an earthquake event, is obtained by averaging all the Mw values for the P and S waves obtained from all stations. This averaging process is carried out to compensate for the effects of using a constant radiation pattern correction^54^. The same procedure was carried out to estimate the moment magnitude of the 2019 Ambon aftershock^55^.

### Focal mechanisms

We used the ISOLA package^19^ to perform moment tensor inversion from the borehole and BMKG seismographic stations (see inverted blue and green triangles in Fig. 1). In order to determine the focal mechanisms, the observed waveforms were pre-processed using a bandpass filter from 0.04 to 0.09 Hz. The data processing stage of the focal mechanism analysis is as follows: (i) convert data from SAC to ASCII format; (ii) input the earthquake hypocenter location (latitude, longitude and depth) and origin time; (iii) station selection; (iv) removal of instrument response, (v) calculation of Green’s function using the discrete-wave number method^56^ to create a synthetic signal based on the 1-D seismic velocity model^57,58^, and (vi) trial a specified range of source depths and origin times; for each trial, moment tensor parameters are computed using least squares minimisation based on the fit between the synthetic and observed waveform. We chose the time window from the origin time to the point where the earthquake signal is no longer detectable above the background noise.

## Supplementary Information

Supplementary Information.

## Data Availability

The new catalog produced by this study is given in Table S2 in the Supplementary Information. The raw data used in this study will be made available through our website (https://www.maipark.com/index.php/en/rdi/maipark_research_data).
