# Supplementary material for: Implications for fault locking south of Jakarta from an investigation of seismic activity along the Baribis fault, northwestern Java, Indonesia
Source: Sci Rep. 2022 Jun 16;12:10143. doi: 10.1038/s41598-022-13896-6 (PMC9203524; doi:10.1038/s41598-022-13896-6)
Supplement: Supplementary file 1 — Supplementary Information. [file 41598_2022_13896_MOESM1_ESM.pdf]

Supplementary information to the manuscript

Implications for Fault Locking South of Jakarta from an Investigation of Seismic Activity along the Baribis Fault, Northwestern Java, Indonesia

S. Widiyantoro, P. Supendi, A. Ardianto, A. W. Baskara, C. A. Bacon, R. Damanik, N. Rawlinson*,

E. Gunawan, D. P. Sahara, Z. Zulfakriza, Y. M. Husni, A. Lesmana

*Corresponding author, nr441@cam.ac.uk

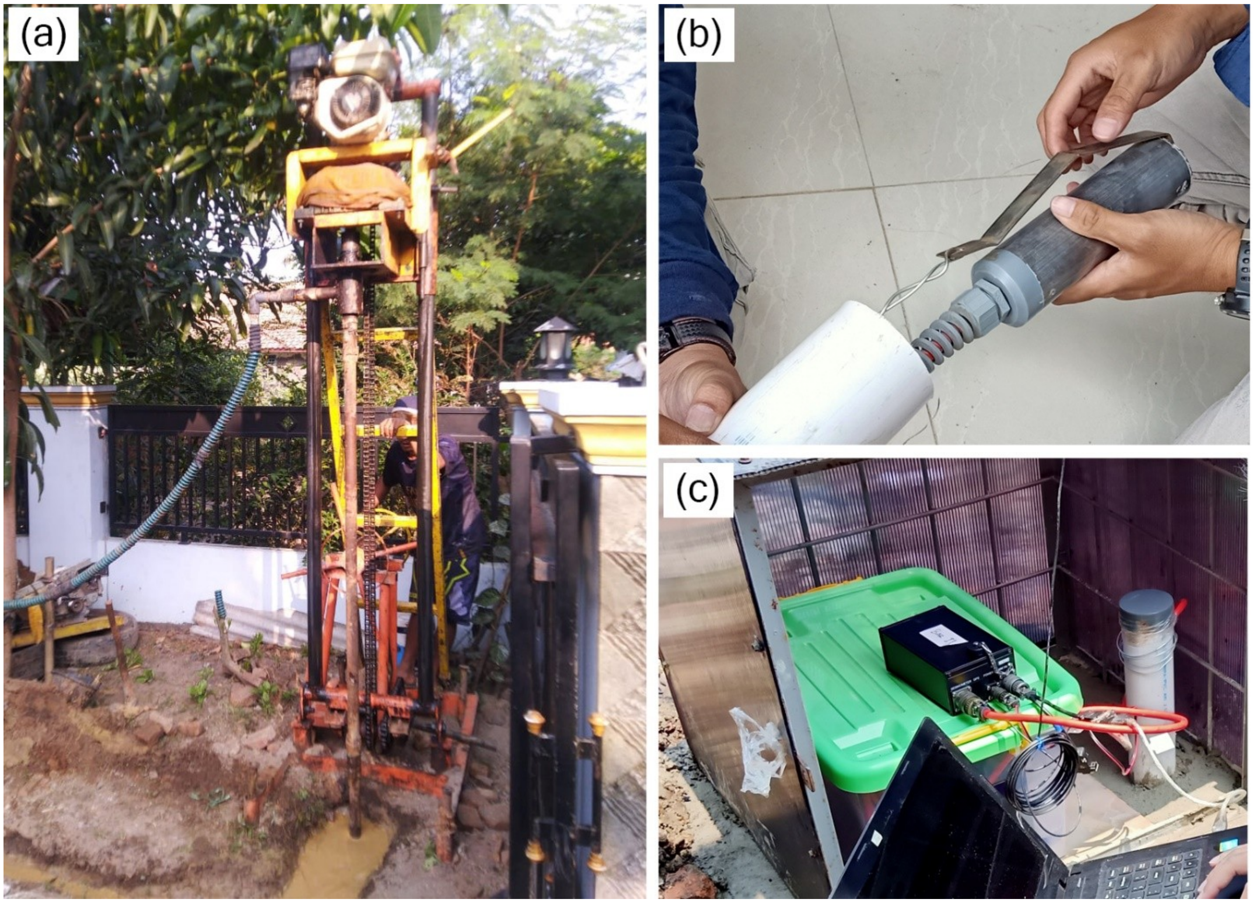

Figure S1. Photographs of instrumentation and example field sites. (a) Drilling prior to seismometer installation at BAR10 in Karawang, (b) C100 wide-band seismometer, and (c) Sri32L Geobit digitizer.

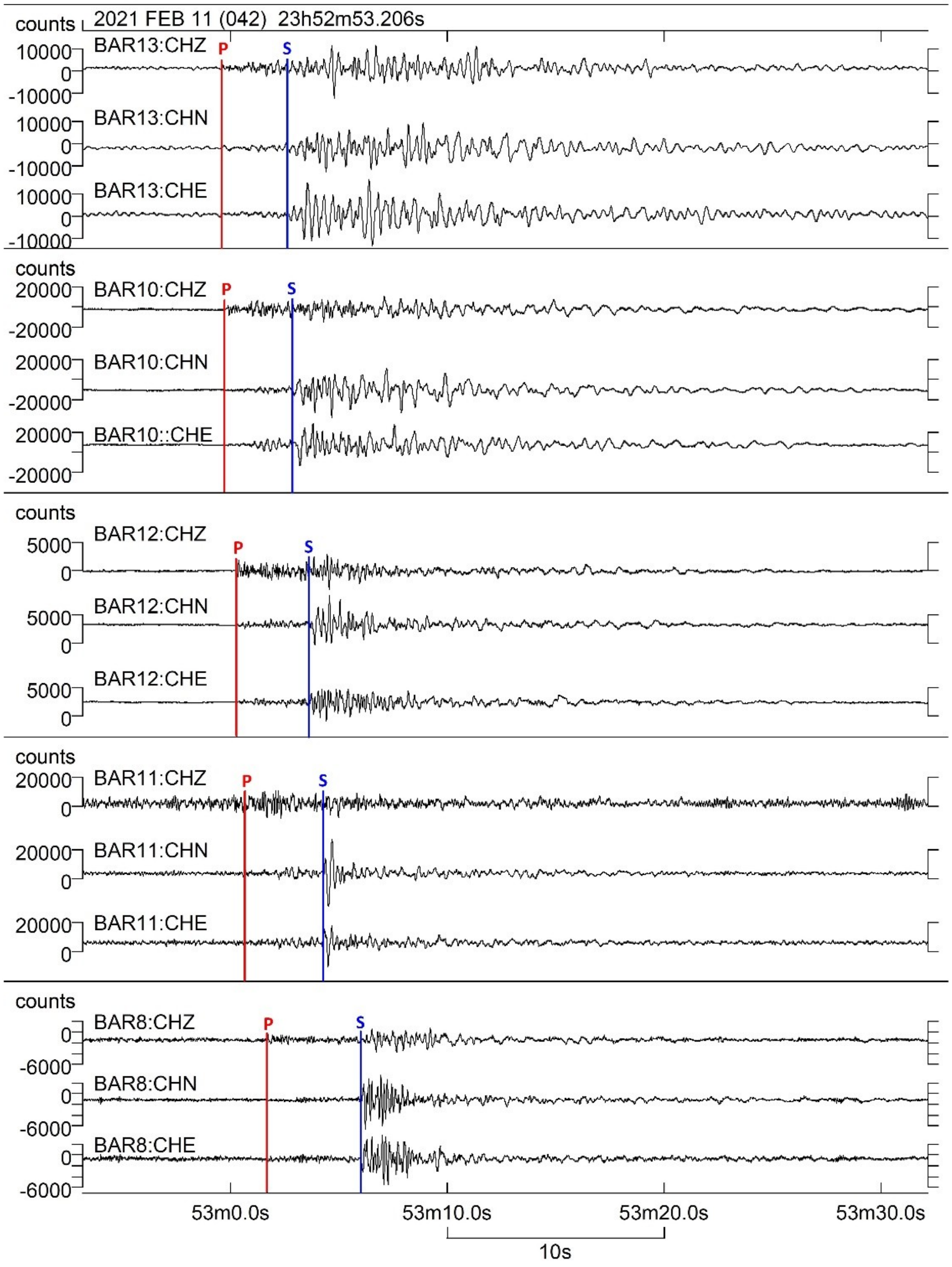

Figure S2. Three-component seismograms showing the February 11, 2021 earthquake (event no. 4 in Fig. 3a) on the Baribis Fault recorded by five borehole seismic stations. Red and blue lines indicate picked P-and S-wave arrivals respectively.

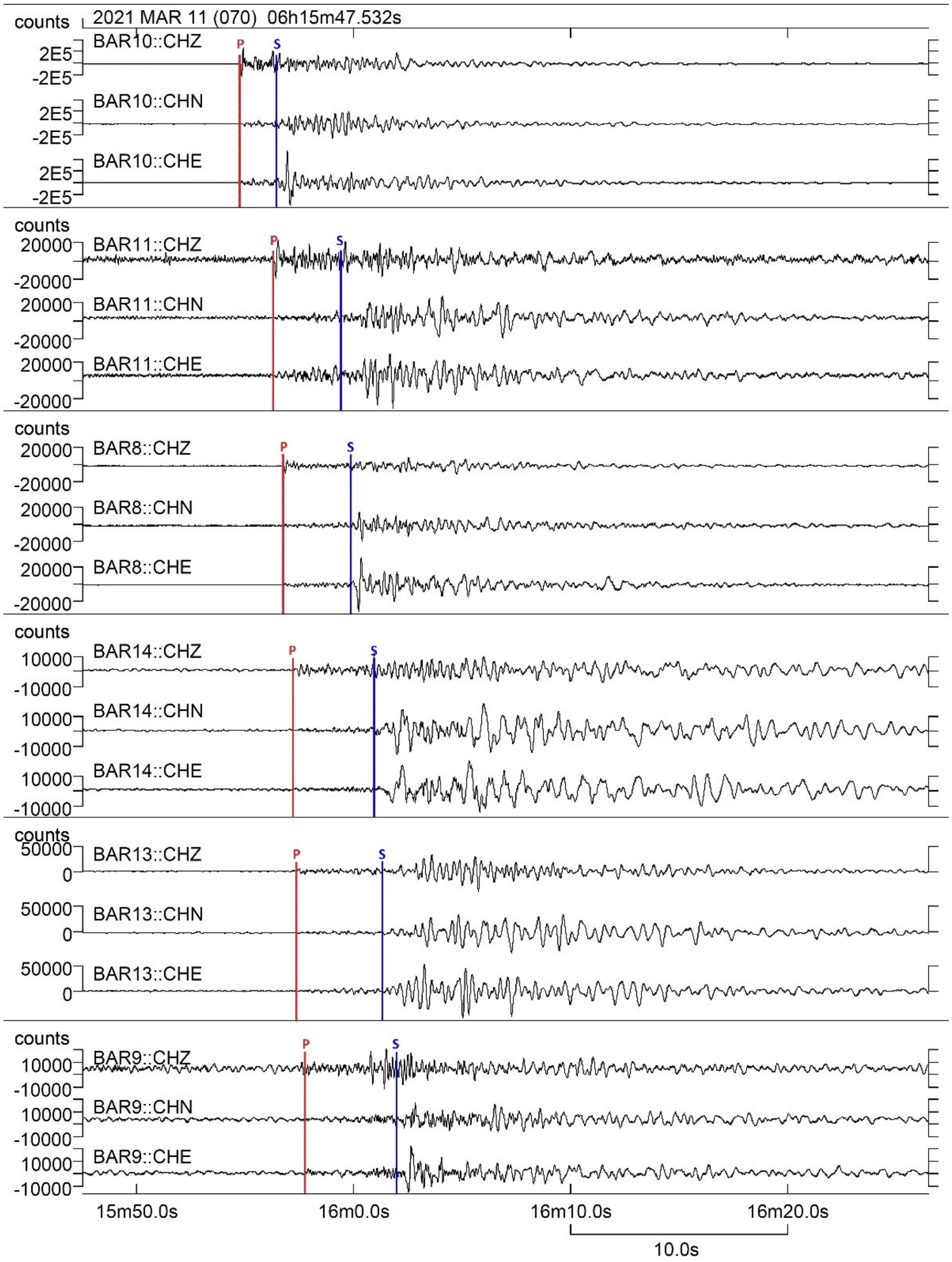

Figure S3. Three-component seismograms showing the March 11, 2021 earthquake (event no. 5 in Fig. 3a) on the Baribis Fault recorded by six borehole seismic stations. Red and blue lines indicate picked P-and S-wave arrivals, respectively.

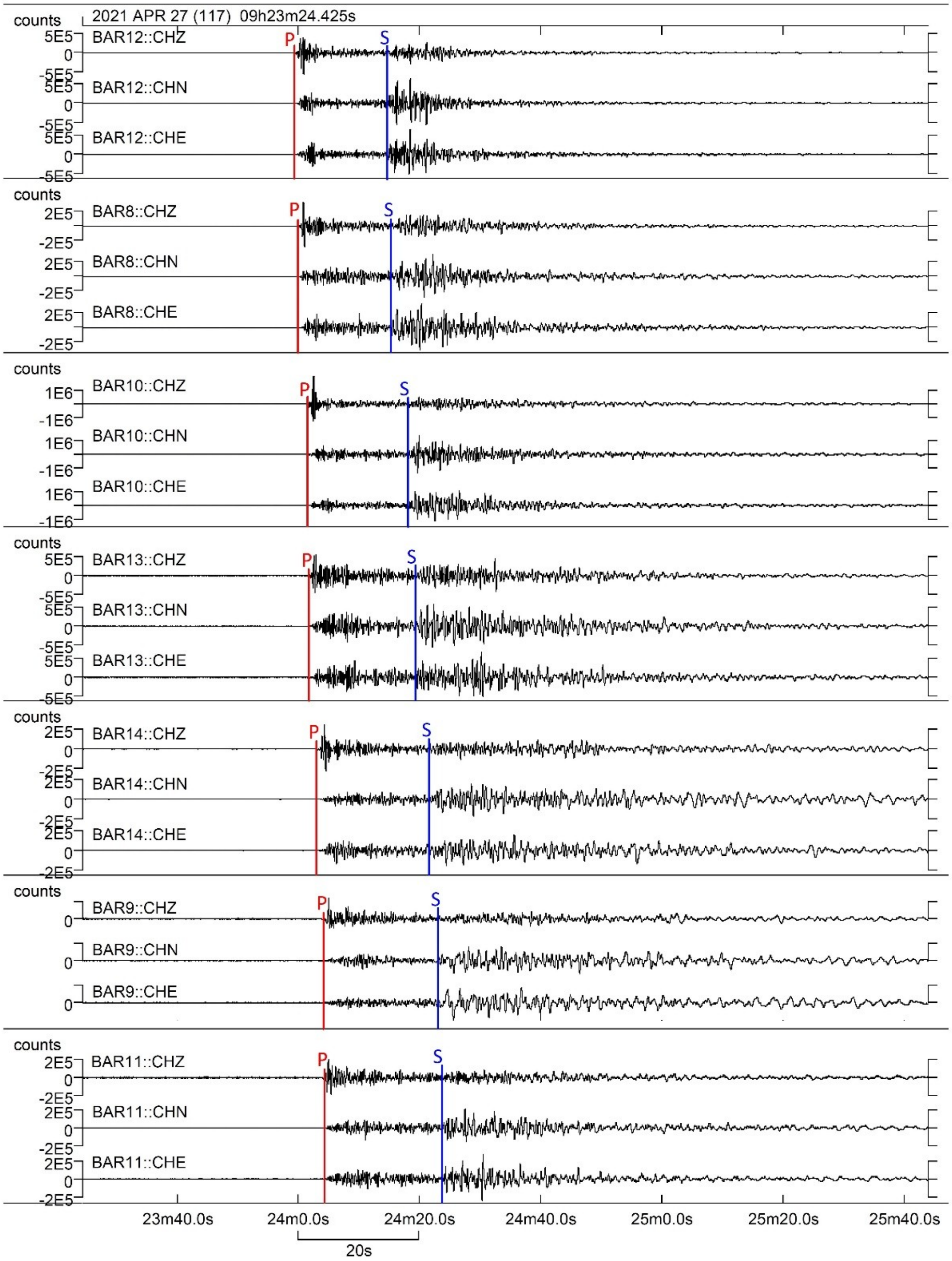

Figure S4. Three-component seismograms showing the April 27, 2021 event south of Java recorded by seven borehole seismic stations. Red and green lines indicate picked P-and S-wave arrivals, respectively.

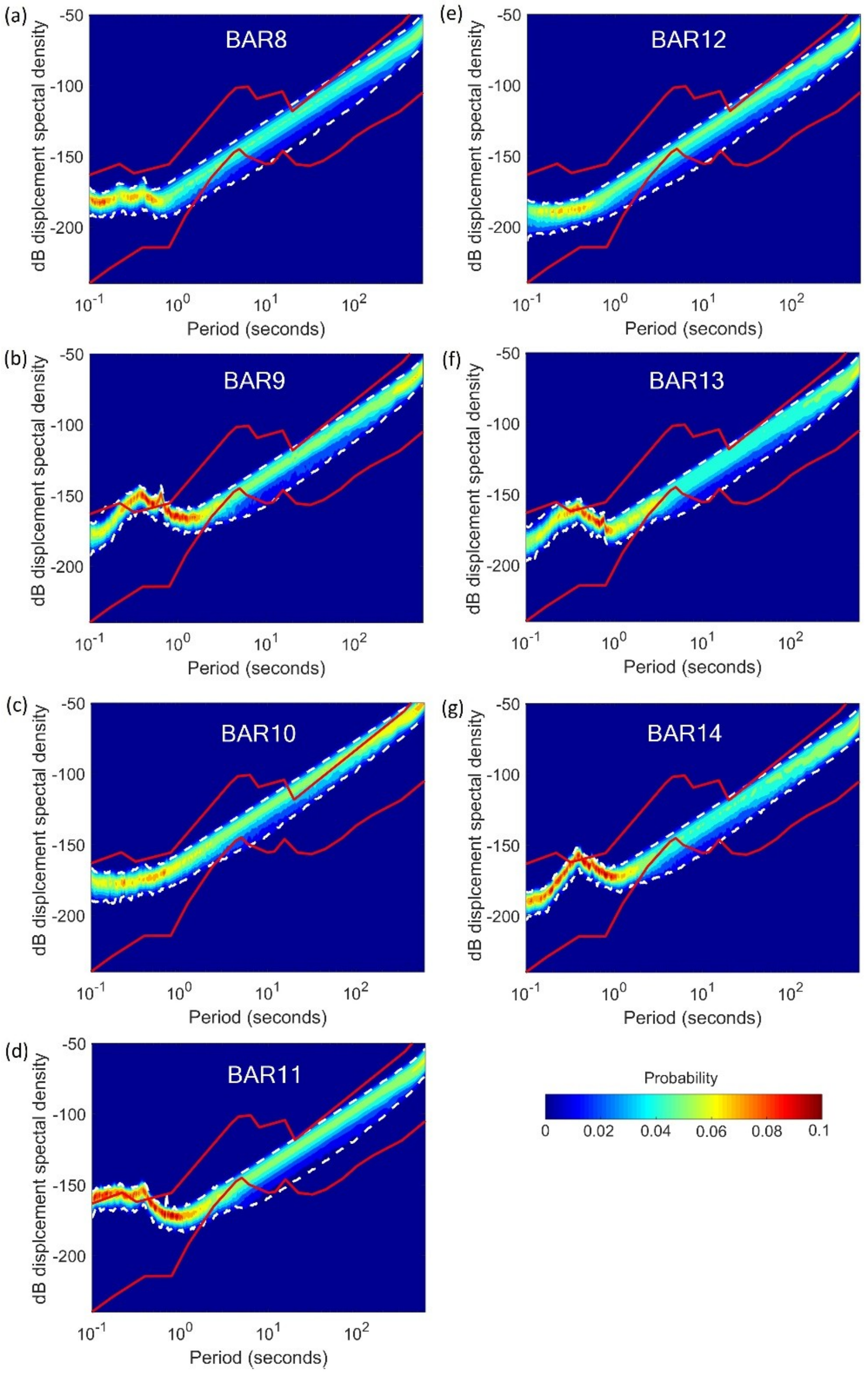

Figure S5. PPSD for stations (a) BAR8, (b) BAR9, (c) BAR10, (d) BAR11, (e) BAR12, (f) BAR13, and (g) BAR14. Each plot was constructed using 4320 power spectral densities (PSDs) from 1 to 30 April 2020. White dashed lines indicate the boundaries of 90 percent of the data

distribution in each period. Red lines depict high- and low-noise models[60]. Station BAR12 had the lowest level of anthropogenic noise, whereas Station BAR11 had the highest. A more diffuse PPSD distribution at Station BAR12 may be related to anthropogenic noise, which has a strong diurnal variation.

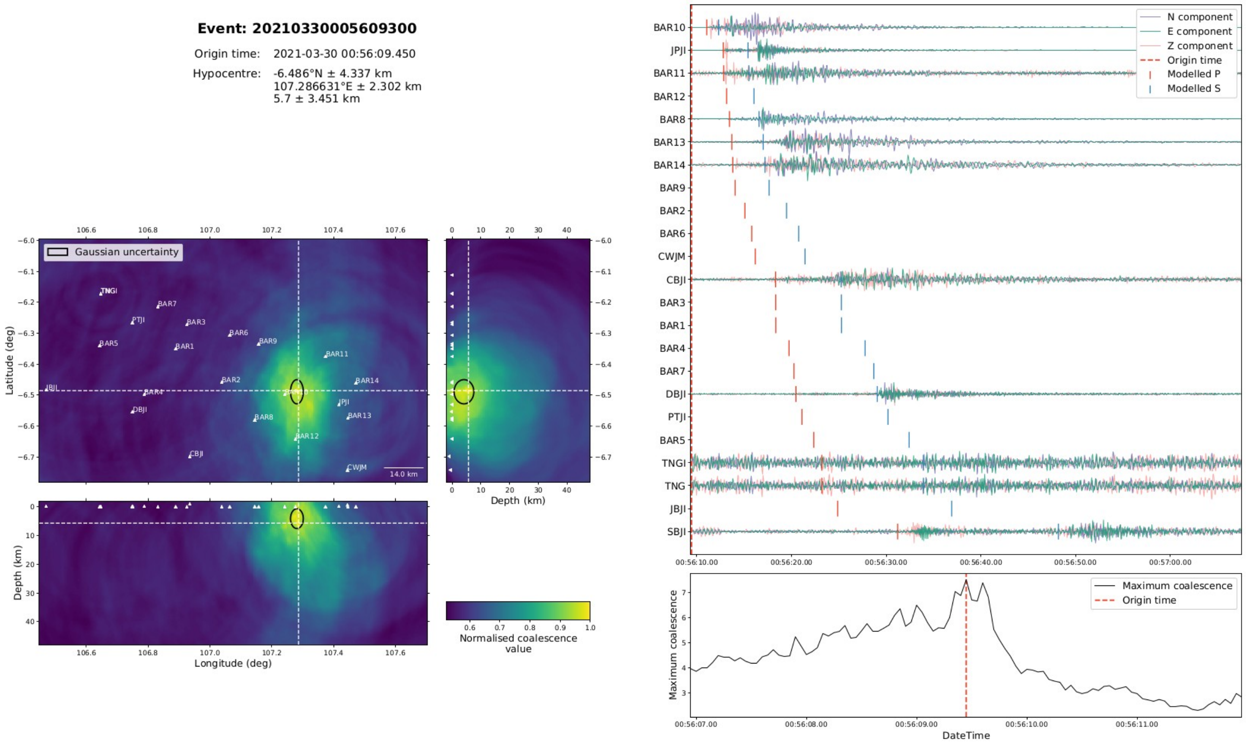

Figure S6: Example of an event detected and located by Quakemigrate as part of this study. Coalescence is a measure of the coherence of a stack of STA/LTA functions. Top right: Observed seismograms with reference model P- and S-wave arrival times superimposed. Bottom Left: Horizontal and vertical slices through the 3-D coalescence function, showing the approximate location of the event. Bottom right: Coalescence as a function of time.

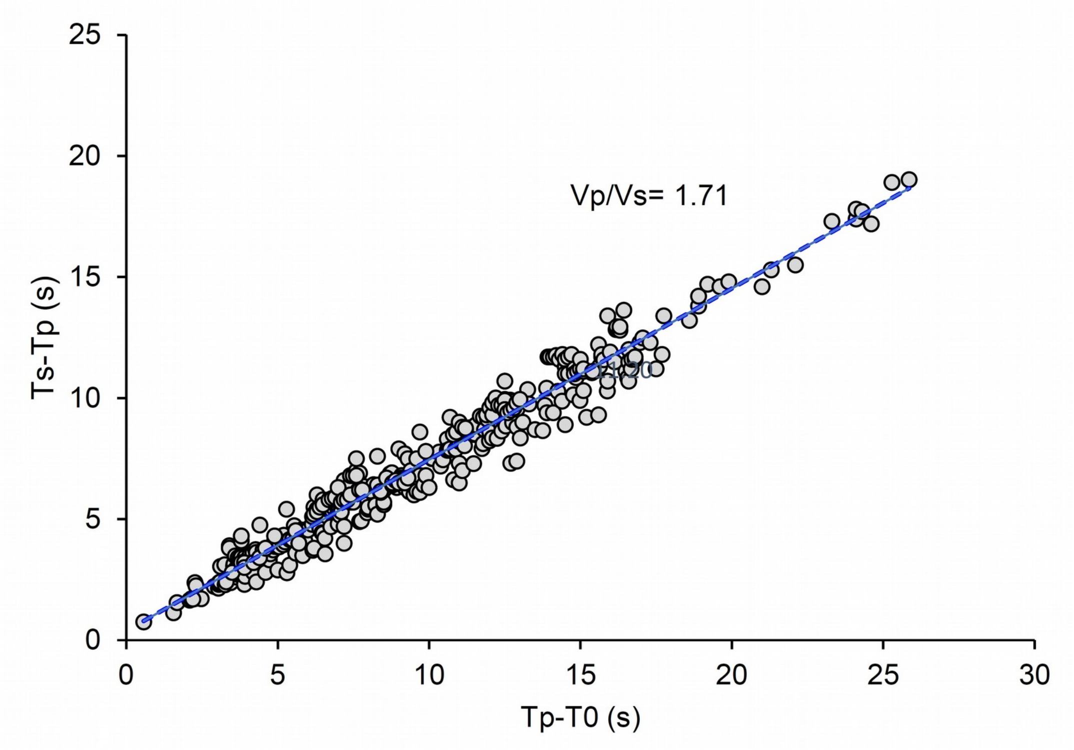

Figure S7. Wadati diagram for the events used in this study. The estimated Vp/Vs ratio is 1.71.

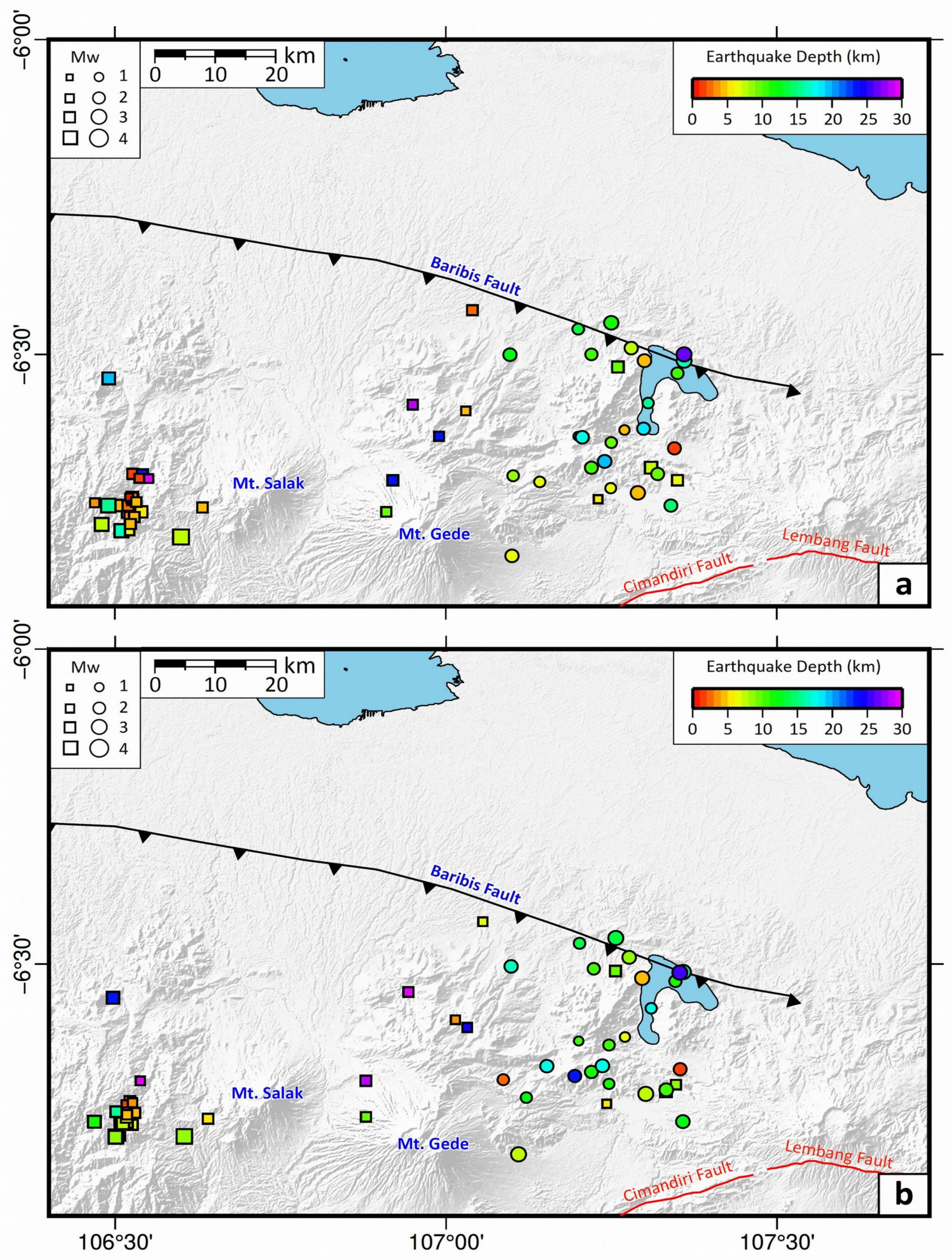

Figure S8. Comparison of epicenters. (a) Before relocation. (b) After relocation using hypoDD. The Generic Mapping Tools (GMT) version 6.0[60] (https://www.generic-mapping-tools.org/) was used to make this figure.

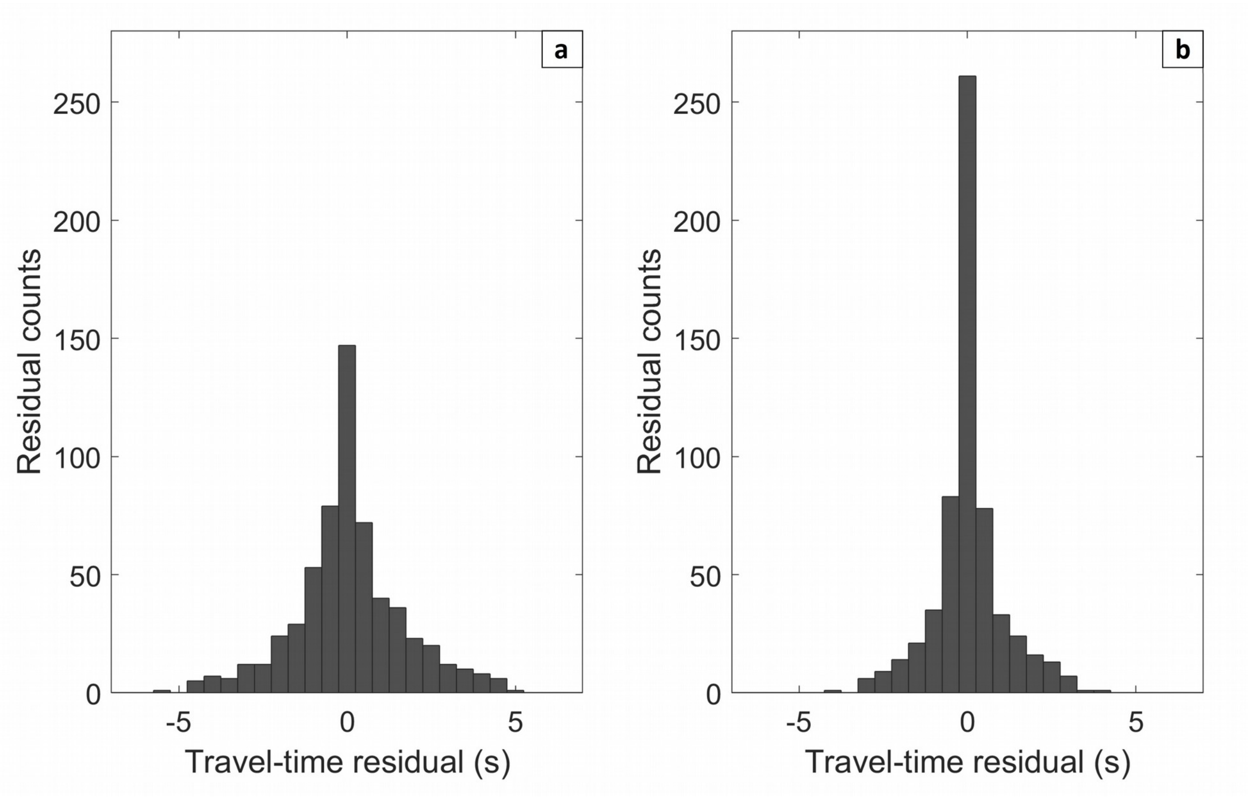

Figure S9. Histograms of relative residuals of event pairs. (a) Before relocation. (b) After relocation using hypoDD.

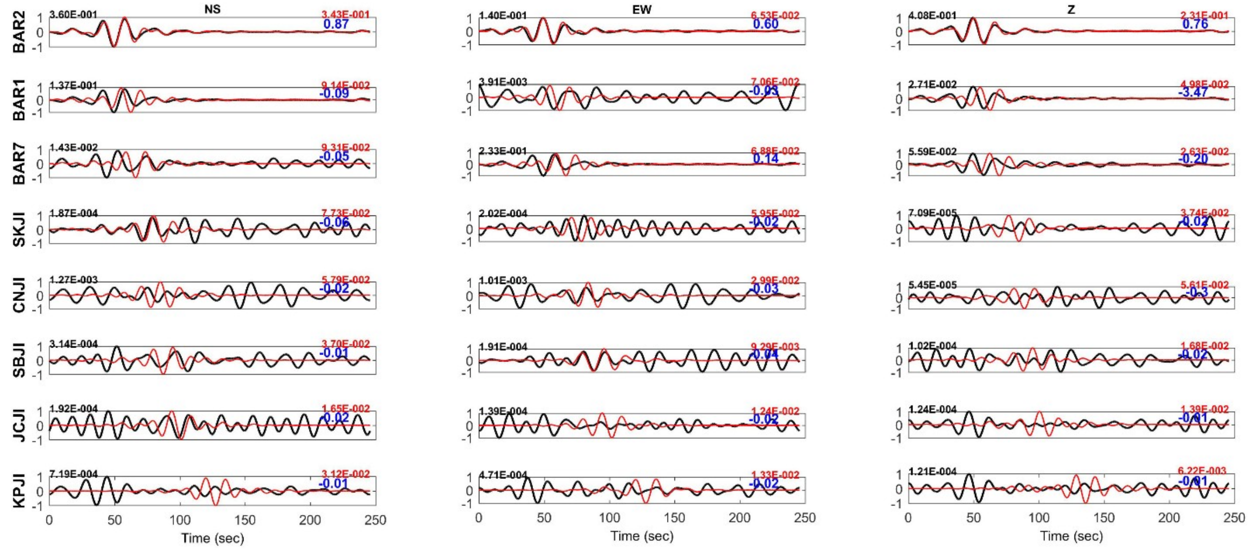

Figure S10. Three-component waveform inversions at stations BAR02, BAR01, BAR07, SKJI, CNJI, SBJI, JCJI, and KPJI of event no. 1 in Fig. 3a that occurred on 10 December 2019. The y-axis denotes the normalized displacement.

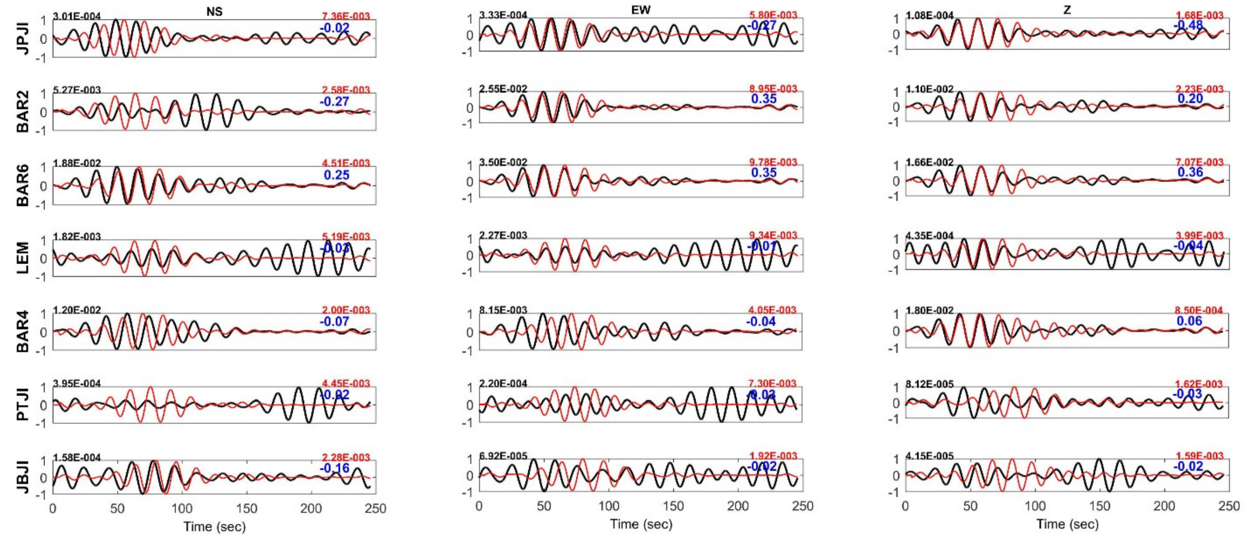

Figure S11. Three-component waveform inversions at stations JPJI, BAR02, BAR06, LEM, BAR04, PTJI, and JBJI of event no. 2 in Fig. 3a that occurred on 29 March 2020. The y-axis denotes the normalized displacement.

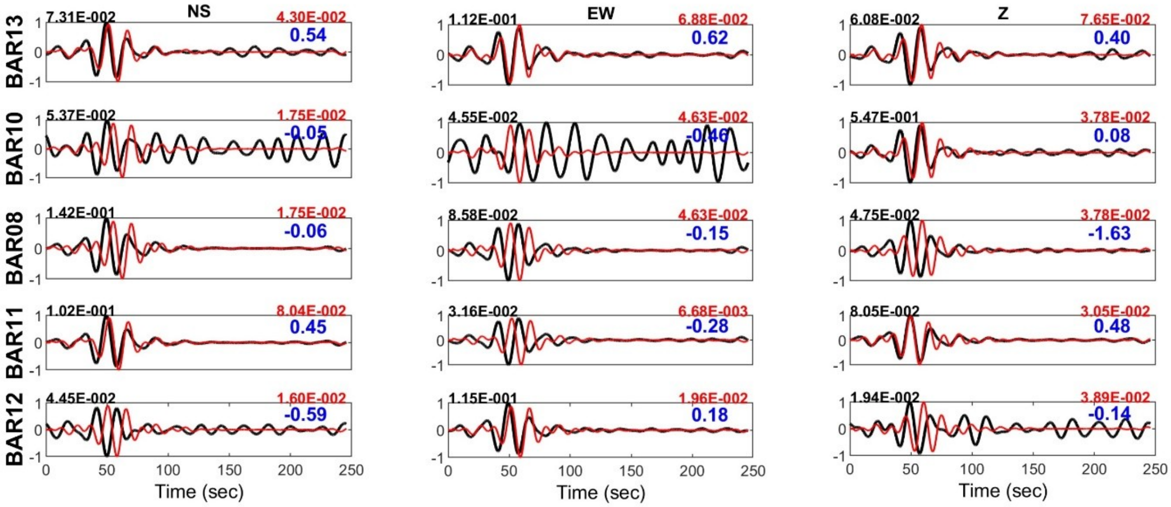

Figure S12. Three-component waveform inversions at stations BAR13, BAR10, BAR08, BAR11, and BAR12 of event no. 3 in Fig. 3a that occurred on 2 February 2021. The y-axis denotes the normalized displacement.

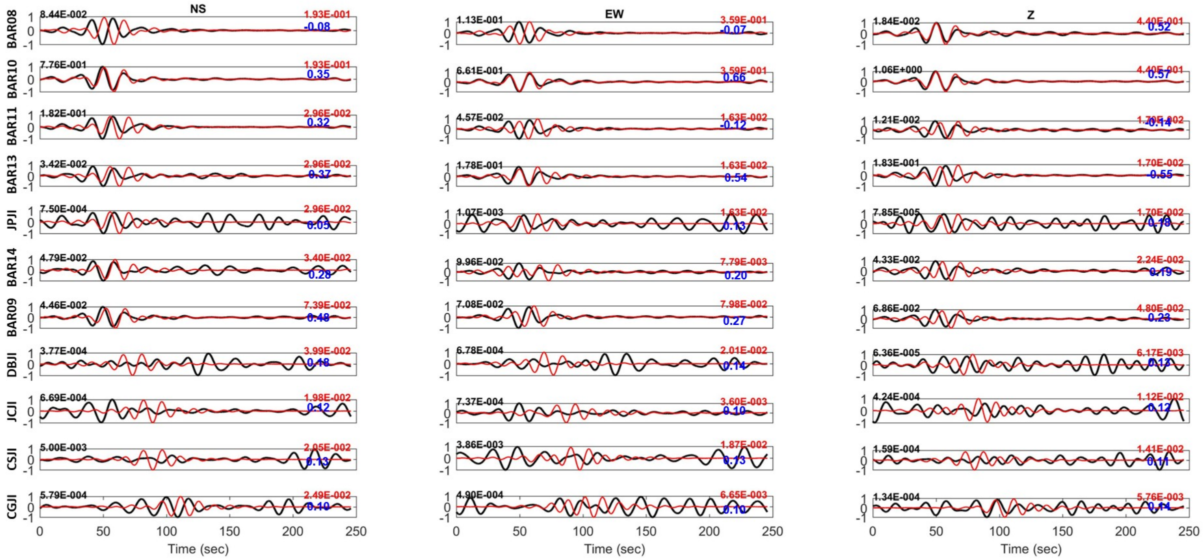

Figure S13. Three-component waveform inversions at stations BAR08, BAR10, BAR11, BAR13, JPJI, BAR14, BAR09, DBJI, JCJI, CSJI, and CGJI of event no. 5 in Fig. 3a that occurred on 11 March 2021. The y-axis denotes the normalized displacement.

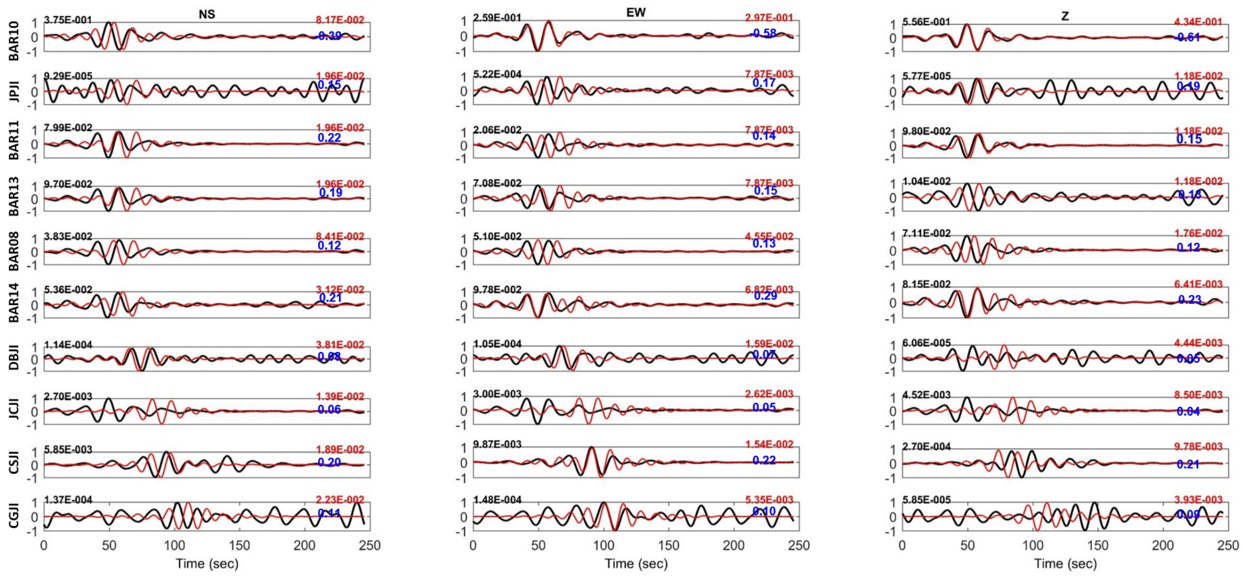

Figure S14. Three-component waveform inversions at stations BAR10, JPJI, BAR11, BAR13, BAR08, BAR14, DBJI, JCJI, CSJI, and CGJI of event no. 6 in Fig. 3a that occurred on 30 March 2021. The y-axis denotes the normalized displacement.

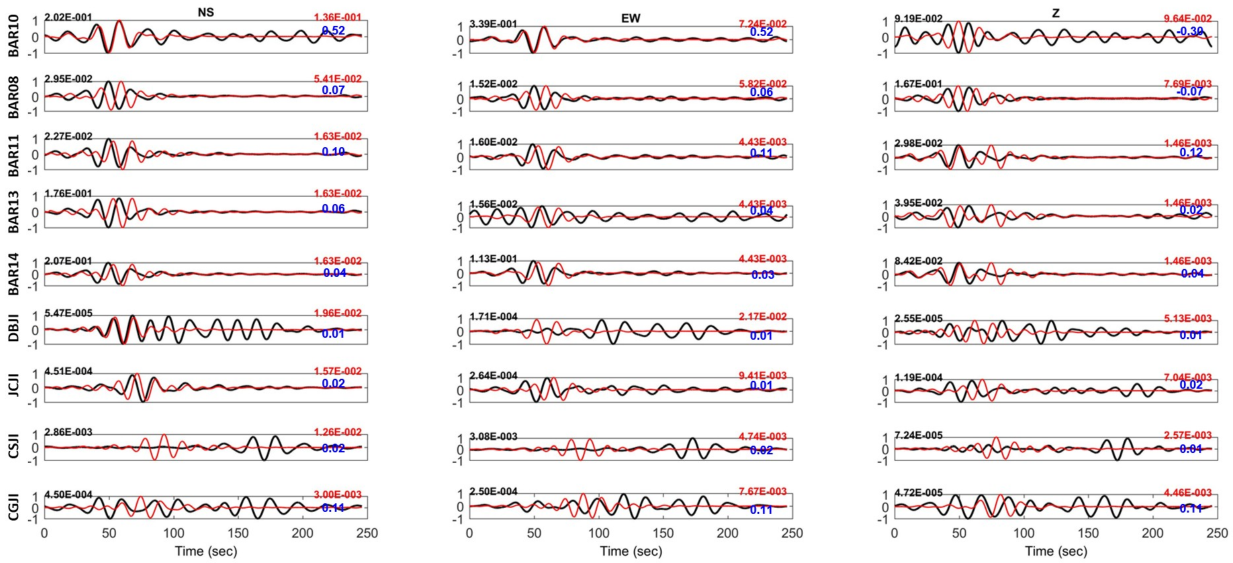

Figure S15. Three-component waveform inversions at stations BAR10, BAR08, BAR11, BAR13, BAR14, DBJI, JCJI, CSJI, and CGJI of event no. 7 in Fig. 3a that occurred on 30 March 2021. The y-axis denotes the normalized displacement.

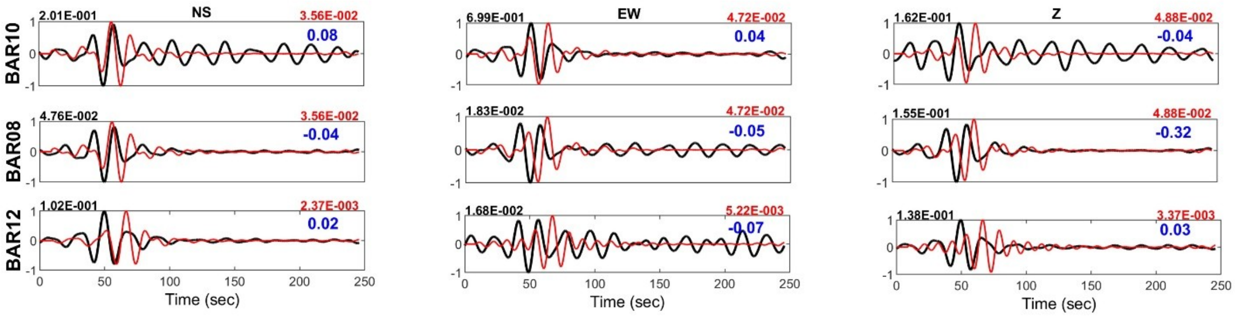

Figure S16. Three-component waveform inversions at stations BAR10, BAR08, and BAR12 of event no. 8 in Fig. 3a that occurred on 1 May 2021. The y-axis denotes the normalized displacement.

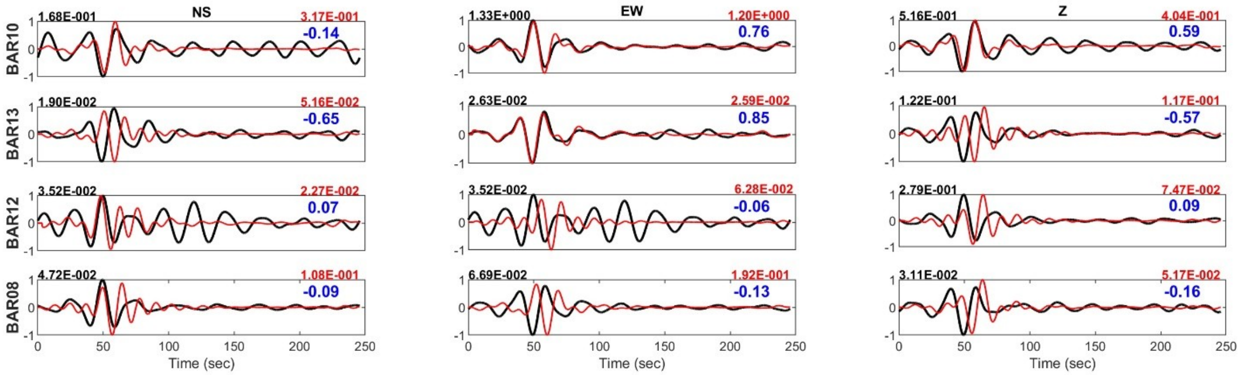

Figure S17. Three-component waveform inversions at stations JPJI, BAR2, BAR6, LEM, BAR3, BAR4, BAR7, PTJI, JBJI, and PBJI of event no. 9 in Fig. 3a that occurred on 29 May 2021. The y-axis denotes the normalized displacement.

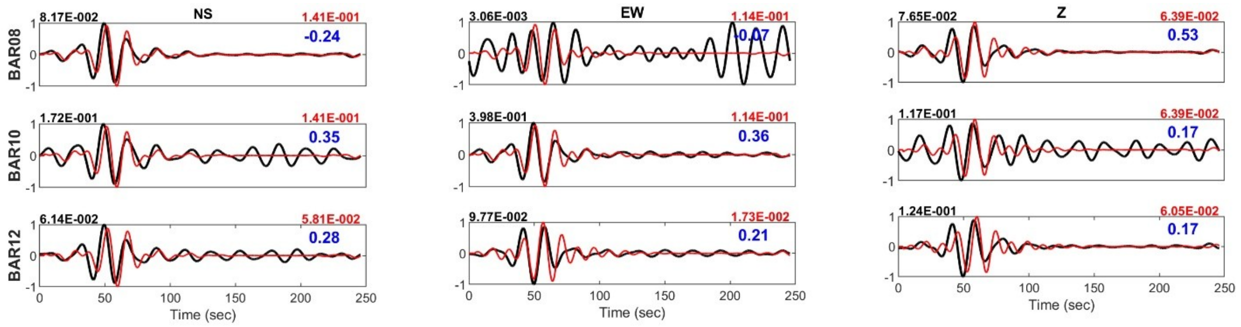

Figure S18. Three-component waveform inversions at stations BAR08, BAR10, and BAR12 of event no. 10 in Fig. 3a that occurred on 10 June 2021. The y-axis denotes the normalized displacement.

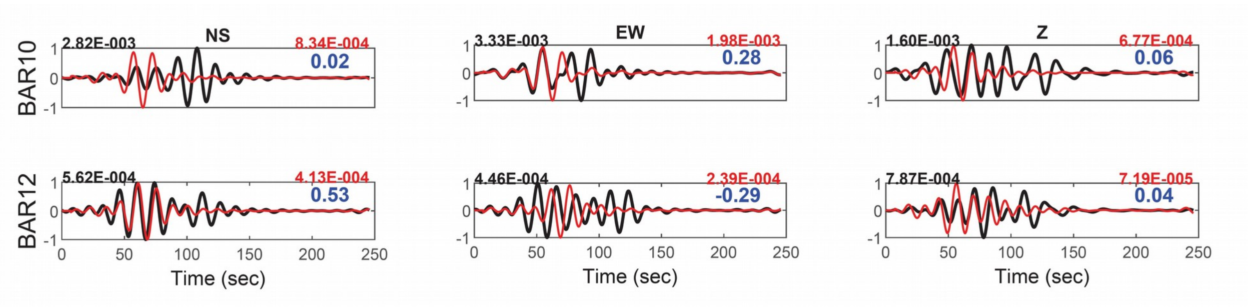

Figure S19. Three-component waveform inversions at stations BAR10 and BAR12 of event no. 11 in Fig. 3a that occurred on 29 June 2021. The y-axis denotes the normalized displacement.

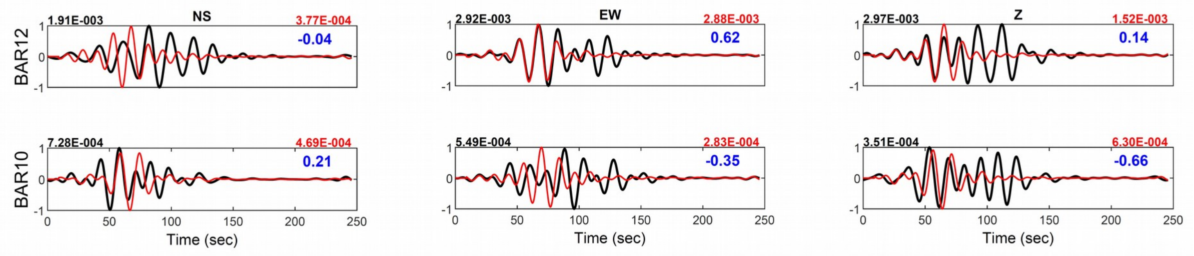

Figure S20. Three-component waveform inversions at stations BAR12 and BAR10 of event no. 12 in Fig. 3a that occurred on 03 July 2021. The y-axis denotes the normalized displacement.

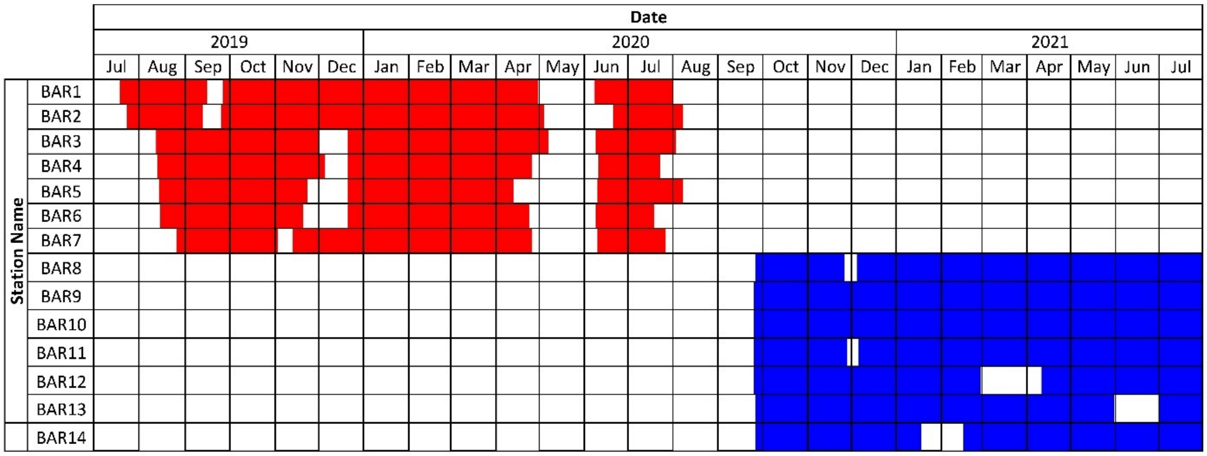

Figure S21. Data acquisition plot for the first year and second year of the borehole station deployment (solid colour indicates when the station listed in the left column is successfully recording). We also note that continuous data from BMKG permanent stations, which spans the entire two year period, are also used in the analysis.

Table S1. The location of seven borehole stations spanning Karawang and Purwakarta, northwestern Java, as deployed in this study.

Station name	Lat (deg)	Long (deg)	Depth (m)
BAR8	-6.58046	107.14495	8.0
BAR9	-6.33426	107.15794	10.0
BAR10	-6.49823	107.24200	10.5
BAR11	-6.37528	107.37389	10.0
BAR12	-6.64193	107.27655	8.0
BAR13	-6.57474	107.44631	9.0
BAR14	-6.46135	107.47226	11.0

Table S2. Catalog of on-land earthquakes based on the results from Damanik et al. (2021)[18] and this study.

Date	Time (UTC)	Lat (deg)	Long (deg)	Depth (km)	Mw	Std. dev. Mw
8/13/2019	14:20:08.2	-6.75052	106.46855	11.483	3.5	0.296
8/19/2019	01:13:12.4	-6.75555	106.52587	6.919	3.0	0.236
8/19/2019	02:44:11.7	-6.75341	106.50696	4.279	3.0	0.157
8/19/2019	06:03:04.3	-6.75038	106.50861	4.017	2.6	0.170
8/19/2019	06:51:37.1	-6.75182	106.51742	4.713	2.9	0.187
8/19/2019	16:10:44.6	-6.71790	106.52241	2.109	3.0	0.162
8/19/2019	22:24:40.4	-6.77369	106.50771	6.060	2.6	0.246
8/19/2019	22:32:21.9	-6.75230	106.51848	4.330	2.9	0.172
8/20/2019	03:57:33.5	-6.58865	107.01401	3.534	2.5	0.417
8/20/2019	20:06:14.8	-6.77386	106.50102	17.325	4.1	0.183
8/20/2019	20:28:54.5	-6.75353	106.51185	7.145	3.4	0.235
8/20/2019	22:29:04.3	-6.72897	106.52141	3.001	3.1	0.160
8/20/2019	22:31:21.3	-6.72872	106.52046	3.433	3.2	0.147
8/21/2019	02:01:20.0	-6.72999	106.51870	3.903	2.8	0.154
8/21/2019	04:24:06.3	-6.74349	106.51716	6.089	3.2	0.209
8/21/2019	04:48:53.6	-6.73667	106.52985	4.324	2.9	0.222
8/21/2019	13:49:56.8	-6.72234	106.51562	1.602	2.1	0.500
8/21/2019	13:52:30.7	-6.73930	106.51807	4.842	2.8	0.179
8/23/2019	04:10:55.0	-6.77518	106.50053	8.805	3.7	0.279
9/7/2019	15:08:57.7	-6.73487	106.50124	15.446	3.1	0.205
10/12/2019	05:25:36.4	-6.54469	106.94299	29.529	2.8	0.278
10/18/2019	14:58:34.0	-6.72051	106.52623	3.038	2.6	0.205
10/29/2019	07:19:46.5	-6.68568	106.53800	29.915	2.5	0.485
10/30/2019	05:17:28.4	-6.60085	107.03179	24.532	2.7	0.252
12/1/2019	05:42:01.5	-6.55384	106.49683	23.234	3.5	0.570
12/10/2019	05:54:19.4	-6.43306	107.05539	6.914	2.5	0.304
1/3/2020	01:59:33.2	-6.68550	106.87890	28.147	3.0	0.22
3/10/2020	10:09:19.5	-6.74575	106.64035	5.600	2.9	0.267
3/10/2020	10:18:02.3	-6.77387	106.60458	8.779	4.3	0.195
3/11/2020	14:41:31.4	-6.70300	107.33203	9.614	3.2	0.331
3/11/2020	14:51:50.6	-6.72219	107.24261	5.210	2.4	0.189
3/15/2020	17:40:40.6	-6.69175	107.34718	8.737	2.8	0.229
3/29/2020	00:38:44.0	-6.51130	107.25570	9.040	3.1	0.265
4/23/2020	05:24:42.4	-6.74295	106.87885	9.720	2.8	0.209
9/19/2020	03:21:09.7	-6.70624	107.30157	7.050	2.9	0.385

9/19/2020	13:25:55.2	-6.66720	107.35358	1.513	2.5	0.251
9/27/2020	09:23:13.4	-7.00648	107.03417	3.464	2.8	0.288
10/30/2020	10:26:46.4	-6.75029	107.35770	12.931	2.7	0.339
10/30/2020	22:25:03.8	-6.70002	107.33247	10.370	2.7	0.373
12/31/2020	16:06:00.7	-6.62874	107.24618	10.269	2.3	0.393
1/3/2021	21:11:01.6	-6.67151	107.21958	11.437	2.5	0.292
2/2/2021	08:21:21.3	-6.51284	107.35848	15.411	2.9	0.349
2/11/2021	23:52:56.5	-6.52756	107.34631	11.740	2.4	0.356
3/5/2021	04:21:57.2	-6.66189	107.23601	17.825	2.7	0.36
3/11/2021	06:15:52.7	-6.52260	107.29630	4.980	2.8	0.245
3/30/2021	00:56:09.3	-6.48966	107.27664	8.799	2.7	0.266
3/30/2021	03:57:28.2	-6.45882	107.25622	13.200	2.9	0.274
5/1/2021	15:47:10.6	-6.46729	107.20168	13.853	2.3	0.353
5/11/2021	13:49:37.8	-6.62250	107.20036	10.663	1.9	0.316
5/16/2021	05:29:16.4	-6.68383	107.08633	2.708	2.3	0.470
5/21/2021	03:49:40.3	-6.61605	107.27004	6.361	2.0	0.282
5/29/2021	17:49:16.8	-6.51383	107.35306	25.692	2.9	0.112
6/5/2021	14:51:59.2	-6.80230	107.10947	7.645	2.9	0.212
6/10/2021	17:05:58.3	-6.50785	107.22304	10.805	2.4	0.503
6/20/2021	16:11:59.3	-6.98246	107.22326	11.333	3.2	0.219
6/29/2021	17:55:15.1	-6.50404	107.09806	16.187	2.6	0.284
6/30/2021	05:52:30.4	-6.71216	107.12130	12.550	2.2	0.234
7/3/2021	21:10:30.3	-6.57050	107.30989	17.126	2.2	0.345
7/7/2021	00:16:46.2	-6.69061	107.24615	11.173	2.1	0.333
7/15/2021	06:27:43.0	-6.67789	107.19450	23.078	2.5	0.289
7/15/2021	06:43:57.4	-6.66241	107.15213	17.726	2.6	0.278

Table S3. Focal mechanism solution data for earthquakes along the Baribis Fault.

Number of event	Date	Time (UTC)	Lat (deg)	Long (deg)	Depth (km)	Mw	Strike (deg)	Dip (deg)	Rake (deg)
1	10 December 2019	05:54:19	-6.433064	107.0554	6.91	2.5	104	71	54
2	29 March 2020	00:38:44	-6.511302	107.2557	9.04	3.1	109	31	85
3	02 February 2021	08:21:26	-6.512837	107.3585	15.41	2.9	93	61	85
4	11 February 2021	23:52:56	-6.527563	107.3463	11.74	2.4	114	62	76
5	11 March 2021	06:15:53	-6.522599	107.2963	4.98	2.8	100	71	69
6	30 March 2021	00:56:09	-6.489662	107.2766	8.8	2.7	111	62	75
7	30 March 2021	03:57:28	-6.458821	107.2562	13.2	2.9	123	53	78
8	01 May 2021	15:47:11	-6.467289	107.2017	13.85	2.3	95	52	70
9	29 May 2021	17:49:17	-6.513834	107.3531	25.69	2.9	110	50	67
10	10 June 2021	17:05:58	-6.507853	107.223	10.81	2.4	95	52	61
11	29 June 2021	17:55:15	-6.50404	107.0981	16.187	2.6	142	53	112
12	03 July 2021	21:10:30	-6.5705	107.3099	17.126	2.2	106	63	53
**Average**							**109**	**57**	**74**

Reference

60. Peterson, J. Observations and modeling of seismic background noise. US Geol. Surv. Open File Rep. https://doi.org/10.3133/ofr93322 (1993).
